# Radiomics of liver MRI predict metastases in mice

**DOI:** 10.1186/s41747-018-0044-7

**Published:** 2018-05-28

**Authors:** Anton S. Becker, Marcel A. Schneider, Moritz C. Wurnig, Matthias Wagner, Pierre A. Clavien, Andreas Boss

**Affiliations:** 1 Institute of Diagnostic and Interventional Radiology, University Hospital Zurich, Raemistrasse 100, 8091 Zurich, Switzerland; 2 Division of Transplantation and Visceral Surgery, University Hospital Zurich, Raemistrasse 100, 8091 Zurich, Switzerland

**Keywords:** Colorectal neoplasms, Computer-assisted image processing, Liver, Magnetic resonance imaging (MRI), Neoplasm micrometastases

## Abstract

**Background:**

The purpose of this study was to investigate whether any texture features show a correlation with intrahepatic tumor growth before the metastasis is visible to the human eye.

**Methods:**

Eight male C57BL6 mice (age 8–10 weeks) were injected intraportally with syngeneic MC-38 colon cancer cells and two mice were injected with phosphate-buffered saline (sham controls). Small animal magnetic resonance imaging (MRI) at 4.7 T was performed at baseline and days 4, 8, 12, 16, and 20 after injection applying a T2-weighted spin-echo sequence. Texture analysis was performed on the images yielding 32 texture features derived from histogram, gray-level co-occurrence matrix, gray-level run-length matrix, and gray-level size-zone matrix. The features were examined with a linear regression model/Pearson correlation test and hierarchical cluster analysis. From each cluster, the feature with the lowest variance was selected.

**Results:**

Tumors were visible on MRI after 20 days. Eighteen features from histogram and the gray-level-matrices exhibited statistically significant correlations before day 20 in the experiment group, but not in the control animals. Cluster analysis revealed three distinct clusters of independent features. The features with the lowest variance were Energy, Short Run Emphasis, and Gray Level Non-Uniformity.

**Conclusions:**

Texture features may quantitatively detect liver metastases before they become visually detectable by the radiologist.

## Key points

- Texture features change systematically in livers with (micro)metastases
- Three clusters of features independently correlated with tumor growth
- Texture features may quantitatively detect hepatic micrometastases before they become visually detectable

## Background

The liver is the primary site of distant hematogenous metastases for cancers of the gastrointestinal tract. Colorectal cancer, for example, the entity being the second highest cause of death in men and women suffering from cancer in the Western world [1], spreads to the liver in about 60% of patients and this is often the reason patients ultimately succumb to their disease [2, 3]. Secondary tumors of the liver, therefore, are still a devastating disease and herald poor prognosis. Fortunately, interventional as well as surgical techniques for treating liver metastases have made tremendous advances in the last few years [4–6]. However, if a curative approach is chosen, preoperative imaging is essential to correctly identify all tumor lesions and avoid leaving behind small tumor nodules in the future liver remnant. Furthermore, in postoperative settings, early and correct diagnosis of recurrent tumor lesions is essential for timely treatment decisions such as salvage chemotherapy or repeat surgery. Hence, today, in most cancer centers, magnetic resonance imaging (MRI) of the liver is an integral part of the workup of patients at risk for liver metastases. Although scan protocols and parameters vary between institutions, T1-weighted and T2-weighted anatomical sequences with high spatial resolution are required [7]. Usually, several contrast-enhanced sequences as well as diffusion-weighted sequence are included as well [8]. Moreover, the advent of intracellular contrast media shows promising results in differentiating metastases from primary liver lesions [9].

Texture analysis is a versatile mathematical technique in the field of image analysis established in the seventies of the past century [10, 11] and expanded in the subsequent decades [12, 13]. In recent years, there has been increasing interest in computing texture features from medical images for quantitative analysis called the “radiomics” approach [14]. In liver, computed tomography texture-based differentiation between normal tissue, benign tumors, and hepatocellular carcinoma has been demonstrated to be possible [15, 16]. Hepatic MRI texture analysis is able to differentiate healthy from cirrhotic liver [17] and even quantify the degree of liver fibrosis [18]. As texture analysis is not only able to detect morphological lesions but also subtle distortions of the tissue architecture, we hypothesized that quantitative texture-based analysis of MRI (a radiomics approach) can identify small niduses of tumor cells earlier than qualitative evaluation by the human eye.

The purpose of this study was to investigate whether any texture features show a correlation with tumor growth before the metastasis can be diagnosed in a human readout based on morphological changes in the images.

## Methods

### Animal experiments

All experiments were carried out in conformity with the local laws and regulations and had been approved by the Cantonal veterinary authorities of Zurich before the trial start. Male C57BL6 mice aged 8–10 weeks, purchased from Harlan (Horst, The Netherlands), were used for all experiments. Animals were kept on a 12:12-h day-night cycle with water and standard rodent chow provided ad libitum. Injections of tumor cells as well as MRI scans were conducted between 8 AM and 12 AM.

### Experimental design

**Fig. 1:**
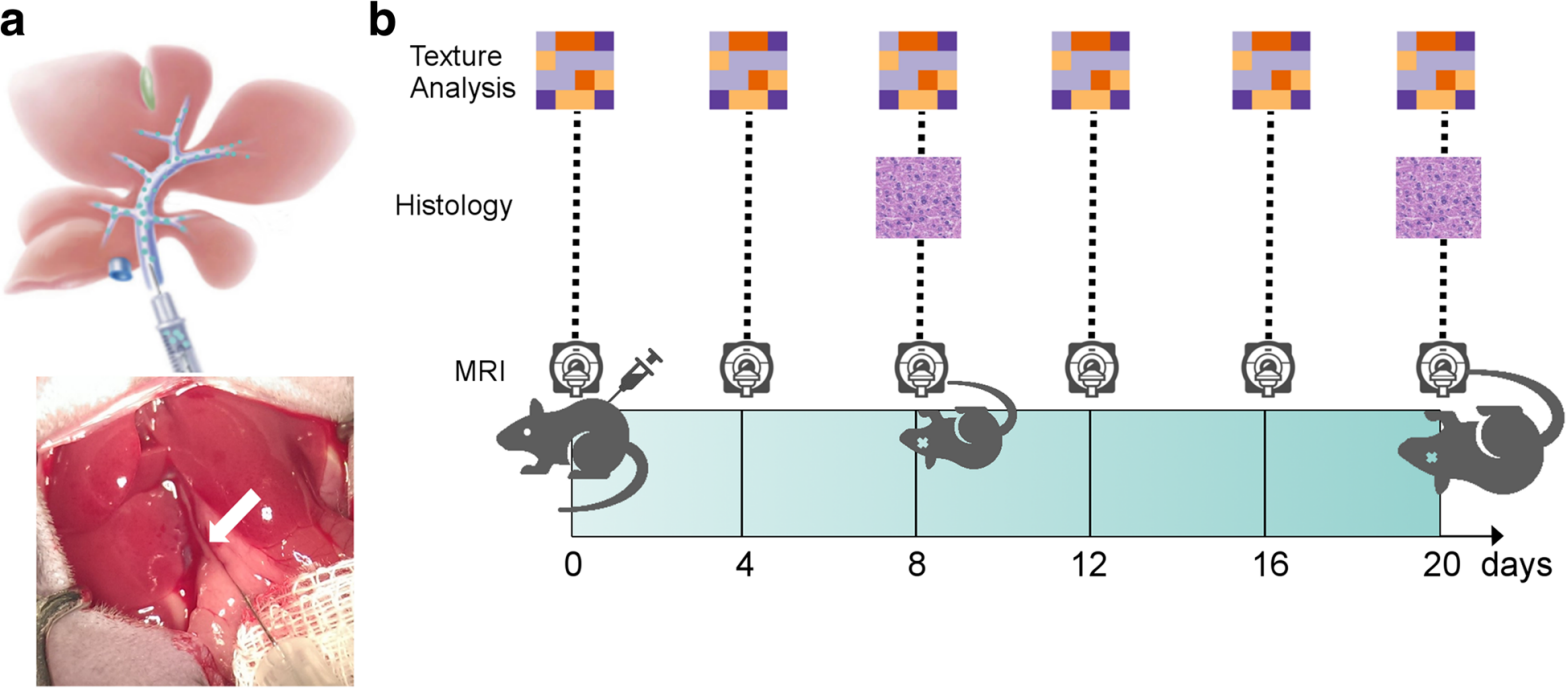
**a** Scheme (*top*) and photography (*bottom*) of the microsurgical intraportal tumor cell or saline (sham) injection. The portal vein being injected is marked with a white arrow. **b** Experimental study design. MRI was performed at baseline (before injection) and at days 4, 8, 12, 16, and 20 post injection. Two animals from the tumor group were sacrificed for histological examination at day 8

Mice were injected with MC-38 tumor cells (*n* = 8) or phosphate-buffered saline (PBS) as controls (sham, *n* = 2). The animals underwent MRI before the injection (baseline) and at days 4, 8, 12, 16, and 20 post injection. The study duration was set after a pilot series (three animals, not included in the current analysis) showed definitely visible liver tumors on MRI after day 20 post injection. At day 8, two animals of the tumor injection group were sacrificed to ensure tumor growth by microscopic examination. At day 20, the remaining animals were sacrificed and the livers harvested for histologic examination. The study design is illustrated in Fig. 1b.

### Tumor cell culture

The murine colon cancer cell line MC-38, syngeneic on a C57BL6 background, was used for the experiments. Cells were cultured in Dulbecco’s modified eagle medium (Life Technologies, Zug, Switzerland) supplemented with 10% fetal bovine serum and 100 U/mL of penicillin and streptomycin and incubated at 37 °C and 5% CO_2_. Cell lines were tested negative for mycoplasma at culture onset (PCR Mycoplasma Test Kit; PromoCell, Heidelberg, Germany). Fur tumor cell injections, cells below passage 10 were harvested by trypsinization, counted with a nucleocounter (Nucleocounter NC-200TM; ChemoMetec A/S, Allerod, Denmark), and prepared in solution of 10^6^ cells/mL PBS.

### Mouse model and surgical procedures

An established model of intraportal injection of syngeneic tumor cells was used for induction of liver tumors as described by Limani et al. [19]. However, in our study, cells were non-selectively injected in all liver lobes. All animal procedures were undertaken by a surgical researcher with extensive experience in advanced experimental microsurgery (MAS). Anesthesia was induced with isoflurane inhalation (Attane, Minrad I, Buffalo, NY, USA) 2–3% mixed with pure oxygen; intraoperative analgesia was administered via subcutaneous application of buprenorphine (0.1 mg/kg body weight). Median laparotomy of approximately 3-cm length was performed after fixation of the animal with tape on a heating pad. The liver was mobilized by cutting the falciform ligament and the membrane between caudate and left lateral lobe with microsurgical scissors. After display of the portal vein, 1 × 10^5^ MC-38 tumor cells, prepared in 100 μL PBS, were injected intraportally with a 29-gauge insulin syringe (12.7-mm needle length; BD Microfine, Franklin Lakes, NJ, USA) as depicted in Fig. 1a. The needle was then removed and hemostasis achieved by gentle pressure with cotton swabs and application of small pieces of Tachosil® (Baxter Inc., Deerfield, IL, USA), if necessary. The abdomen was closed with two layered continuous sutures with 5-0 prolene. Mice were allowed to recover on a warmed heating pad; food and water were provided 1 h after the operation. Postoperative analgesia with buprenorphine was administered via drinking water for three days. Livers were harvested at indicated time points (see below) under anesthesia and analgesia as described above. After re-opening of median laparotomy, animals were euthanized by bilateral pneumothorax and trans-section of inferior vena cava and aorta. Organs were harvested quickly and immediately stored in 4% formaldehyde in PBS (% volume/volume).

### Histological examination

After storage in 4% formaldehyde for 48 h, whole livers were embedded in paraffin blocks in a position resembling transversal slices of the MRI. The whole block was afterwards cut with a cryotome and representative histological slides containing liver and tumor tissue prepared at every millimeter. Slides were colored with hematoxylin-eosin (H&E) staining according to standard protocols.

All slides were afterwards scanned with a NanoZoomer XR Digital slide scanner C12000 (Hamamutsu, Japan) and analyzed with the freely available software NDP.view2 (Version 2.6.13, Hamamutsu, Japan). Each slide was separately scanned for tumor lesions in the whole depicted liver parenchyma. Area (μm^2^) and perimeter (μm) of each tumor lesion were measured with the Freehand Region of Interest Tool of the NDP.view2 software, as well as the total amount of detected tumor lesions in all slides of each individual animal calculated.

### MRI

All mice underwent abdominal MRI examinations in a dedicated small animal 4.7-T scanner (Bruker 4.7-T PharmaScan 47/16 US, Bruker BioSpin MRI GmbH, Ettlingen, Germany) under general anesthesia with isoflurane (Attane; Minrad I, Buffalo, NY; 2–3% mixed with pure oxygen). Spin excitation and signal reception were performed with a linearly polarized ^1^H whole-body mouse coil. The mice were placed in supine position in the scanner bed and kept warm with a pad circulating a continuous supply of warm water during continuous anesthesia. MRI was performed during free breathing with respiratory control. A T2-weighted rapid acquisition with refocused echoes sequence was acquired in transverse orientation with the following parameters: echo time = 19 ms; repetition time = 1000 ms; echo-train length = 4; pixel bandwidth = 310 Hz/pixel; excitations = 2; matrix size = 192 × 192; field of view = 30 × 30 mm; slice thickness = 1.5 mm. The images at each time point were evaluated qualitatively by two independent readers (ASB, AB) for visibility of metastases. From the visible metastases at day 20, in each mouse, one metastasis not yet visible at day 16 was chosen, with easily reproducible slice position due to anatomical landmarks.

### Signal-to-noise and contrast-to-noise evaluation

The signal-to-noise ratio (SNR) was determined as follows:

$$ SNR=\frac{SI\ast \sqrt{2}}{noise} $$

where SI is the signal intensity in either the liver parenchyma and noise representing the standard deviation in the background (air) measured in the corner of the image outside areas of artefacts. Contrast-to-noise ratio (CNR) was defined as:

$$ CNR=\frac{S{I}_{meta}-S{I}_{liver}}{noise} $$

With SI_meta_ and SI_liver_ meaning the signal intensity in the metastases and liver parenchyma, respectively.

### Texture analysis

**Fig. 2:**
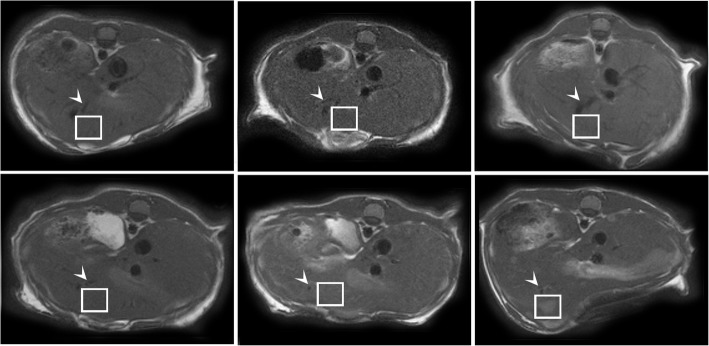
Sample slices of an animal of the experiment group. The metastases are well delineated after 20 days but not definitely visible beforehand. The three vessel branches near the ROI (*arrowhead*) serve as an anatomical landmark to analyze the same volume of liver tissue in the images before day 20, when the metastasis is not visible yet

Texture analysis was performed with an in-house developed MATLAB routine (v2016, The MathWorks Inc., Natick, MA, USA) by the same readers in consensus. On a single day-20 slice (acquired at post-injection day 20), a quadrangular 32 × 32 pixel region of interest (ROI) was placed in the liver, encompassing a distinct metastasis as illustrated in Fig. 2. The ROI was manually copied to the same slice at the four earlier time points at the same position, with the help of anatomical landmarks if the metastasis itself was not visible. From the two control animals (sham), two and three slices were analyzed in order to yield five data points and reasonable confidence intervals. Before texture analysis, ROI contents were normalized between the mean and three standard deviations to minimize intra- and inter-scanner fluctuations in texture analysis [20].

Thirty-two texture features were computed: four first-order and 28 higher-order features analogous to those described by Becker et al. [21] and Vallières et al. [22], as summarized in Table 1. The first order features were computed directly from the histogram of the original image, whereas the higher order features were obtained from the gray-level co-occurrence matrix (GLCM), the gray-level run-length matrix (GLRLM), or the gray-level size zone matrix (GLSZM). Albeit some of these features have “intuitive names” (“intuitive” in this context meaning easily distinguishable by the human observer), none resemble or describe any intuitive patterns [11]. The mathematical definition of the respective features can be found in the works by Haralick et al. for the GLCM [23], Mary M. Galloway for the GLRLM [10], and Thibault et al. for the GLSZM [13].

**Table 1:** Texture features used in the present study

Primary	Higher order		
Histogram	Gray-level co-occurrence matrix (GLCM)	Gray-level run-length matrix (GLRLM)	Gray-level size-zone matrix (GLSZM)
Variance	Contrast	Short run emphasis (SRE)	Small zone emphasis (SZE)
Skewness	Correlation	Long run emphasis (LRE)	Large zone emphasis (LZE)
Kurtosis	Energy	Gray-level non-uniformity (GLN)	Gray-level non-uniformity (GLN)
Entropy	Homogeneity	Run length non-uniformity (RLN)	Zone-size non-uniformity (ZSN)
		Run percentage (RP)	Zone percentage (ZP)
		Low gray-level run emphasis (LGRE)	Low gray-level zone emphasis (LGZE)
		High gray-level run emphasis (HGRE)	High gray-level zone emphasis (HGZE)
		Short run low gray-level emphasis (SRLGE)	Small zone low gray-level emphasis (SZLGE)
		Short run high gray-level emphasis (SRHGE)	Small zone high gray-level emphasis (SZHGE)
		Long run low gray-level emphasis (LRLGE)	Large zone low gray-level emphasis (LZLGE)
		Long run high gray-level emphasis (LRHGE)	Large zone high gray-level emphasis (LZHGE)
			Gray level variance (GLV)
			Zone size variance (ZSV)

### Statistical analysis

Statistical analysis was performed using the “R” software (v3.3.1., The R Foundation for Statistical Software, Vienna, Austria). Graphs were generated using “ggplot2” [24]. All features were evaluated over the whole time course with a linear model/Pearson correlation test. A *p* value < 0.05 was considered statistically significant. The *p* value was not corrected for multiple comparisons due to the exploratory nature of the analysis. However, the number of features was reduced with the following three steps: 1 = significantly changing features in the sham group were excluded from the final set; 2 = features were examined for redundancy by co-correlation testing (Pearson) and hierarchical clustering to determine groups of independently changing features; and 3 = from each cluster, the feature with the smallest variance was selected as the most representative one.

## Results

### Study procedures

**Fig. 3:**
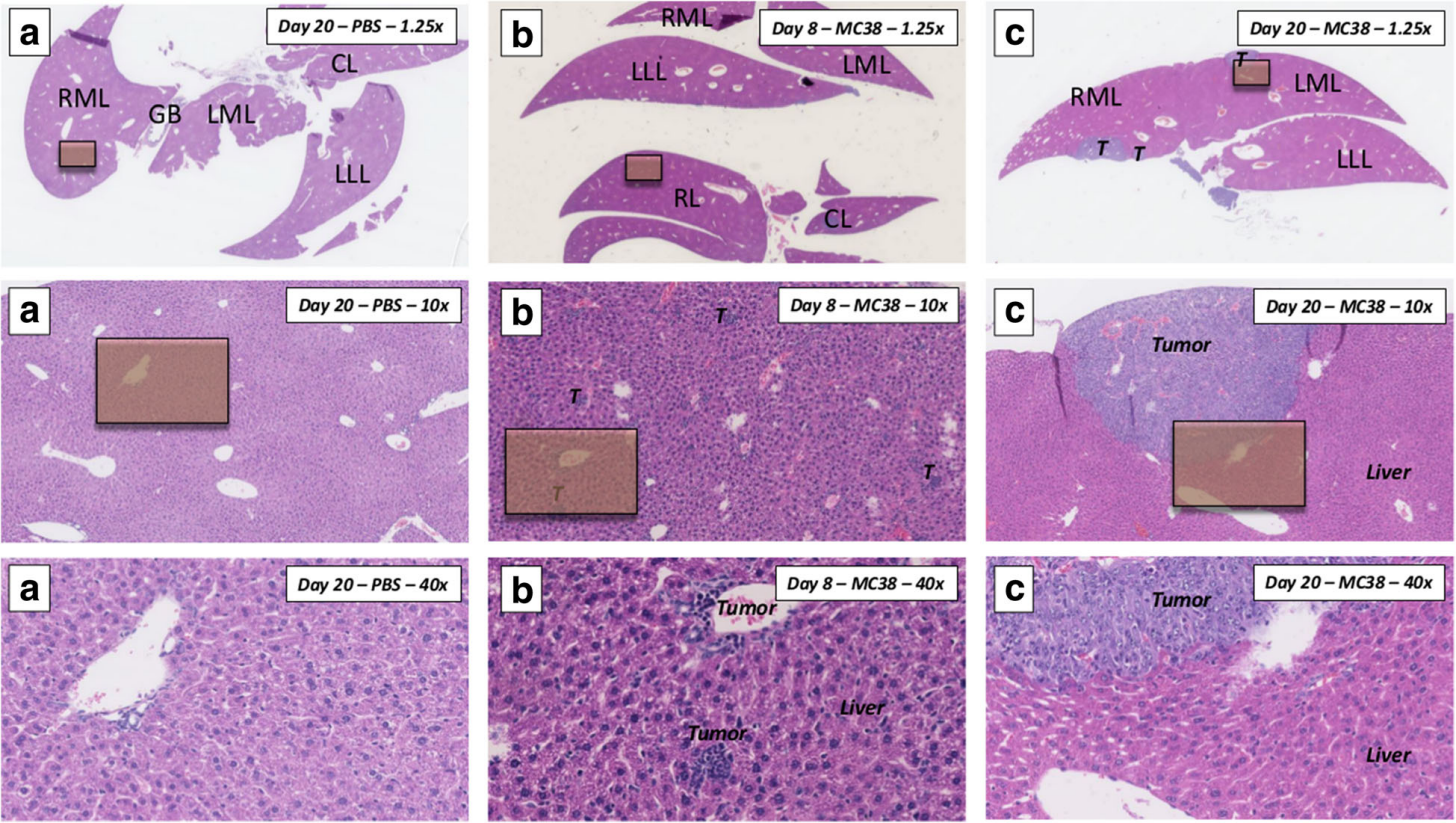
Representative histological images of mouse livers (H&E staining). RML right median lobe, GB gall bladder, LML left median lobe, LLL left lateral lobe, CL caudate lobe, RL right inferior and superior lobe. *Orange squares* mark the area of 40× magnification for the respective images below. **a** Overview of mouse liver with control PBS injection, harvested at day 20. Overview in 1.25× magnification shows complete transversal section of the liver covering RML, GB, LML, LLL, and the bifid CL. 10× magnification and 40× magnification show intact liver parenchyma without any signs of tumor invasion. **b** Overview of a mouse liver harvested on day 8 after non-selective intraportal injection of syngeneic MC38 tumor cells. While no tumor can be detected macroscopically and in the overview of the specimen, 10× and 40× magnification reveal small nests of intraparenchymal and paravascular tumor cells, accompanied by infiltrating leukocytes. **c** Overview of mouse liver harvested on day 20 after non-selective intraportal injection of syngeneic MC38 tumor cells. Multiple tumor nodules can be appreciated already at a macroscopic level in all liver lobes

The intraportal tumor cell and sham injections were performed successfully and without any complications. MRI scans before injection (baseline) and at days 4, 8, 12, 16, and 20 after injection of MC38 tumor cells were completed successfully. Presence of tumor cells in the liver parenchyma was confirmed histologically after eight days in two mice, which were sacrificed for this purpose (Fig. 3). At post-injection day 20, T2-weighted images showed well visible hyperintense liver tumors in all six remaining mice (Fig. 2, bottom right).

### Morphological evaluation

On MRI, the mice exhibited a median of four metastases on day 20 (range = 3–11). In each animal, there was at least one metastasis near an anatomic landmark which was reliably depicted on all days and thus suitable for texture analysis. On histology on day 8, the median circumference was 0.173 mm (interquartile range [IQR] = 0.137–0.205 mm) corresponding to an area of 0.001812 mm^2^ (IQR = 0.001243–0.002513 mm^2^); on day 20, the circumference had grown to 3.57 mm (IQR = 2.19–9.27 mm) which corresponds to an area of 0.61 mm^2^ (IQR = 0.24–5.10 mm^2^).

### Signal-to-noise and contrast-to-noise ratios

SNRs were (mean ± standard deviation) 28.98 ± 6.52 (day 0), 23.71 ± 9.90 (day 4), 28.38 ± 6.98 (day 8), 26.44 ± 6.23 (day 12), 26.72 ± 7.32 (day 16), and 28.08 ± 8.15 (day 20). CNR of metastases on day 20 was 6.88 ± 4.63.

### Texture analysis

Texture features were computed successfully for all time points and animals. Linear fitting revealed significant correlation in 18 features in the experiment group, as follows (full names in Table 1):

- First order: Kurtosis
- GLCM: Contrast, Correlation, Energy, Homogeneity
- GLRLM: SRE, LRE, GLN, RLN, RP, LRHGE
- GLSZM: SZE, LZE, GLN, ZSN, ZP, SZHGE, LZHGE

Correlation coefficients and *p* values are summarized in Table 2.

**Table 2:** Correlating features with Pearson correlation coefficients (R) and *p* values

Feature	R	*p* value
Variance	0.204	0.194
Skewness	0.198	0.210
Kurtosis	0.411	0.007
Contrast (GLCM)	− 0.361	0.019
Correlation (GLCM)	0.393	0.010
Energy (GLCM)	0.392	0.010
Homogeneity	0.432	0.004
Entropy (GLCM)	− 0.014	0.930
SRE (GLRLM)	− 0.394	0.010
LRE (GLRLM)	0.410	0.007
GLN (GLRLM)	0.419	0.006
RLN (GLRLM)	− 0.398	0.009
RP (GLRLM)	− 0.406	0.008
LGRE (GLRLM)	− 0.103	0.516
HGRE (GLRLM)	− 0.129	0.417
SRLGE (GLRLM)	− 0.110	0.486
SRHGE (GLRLM)	− 0.286	0.067
LRLGE (GLRLM)	− 0.089	0.575
LRHGE (GLRLM)	0.407	0.008
SZE (GLSZM)	− 0.447	0.003
LZE (GLSZM)	0.386	0.011
GLN (GLSZM)	0.386	0.011
ZSN (GLSZM)	− 0.445	0.003
ZP (GLSZM)	− 0.428	0.005
LGZE (GLSZM)	− 0.092	0.561
HGZE (GLSZM)	− 0.182	0.248
SZLGE (GLSZM)	− 0.117	0.462
SZHGE (GLSZM)	− 0.345	0.025
LZLGE (GLSZM)	− 0.051	0.750
LZHGE (GLSZM)	0.383	0.012
GLV (GLSZM)	− 0.269	0.085
ZSV (GLSZM)	− 0.086	0.589

**Fig. 4:**
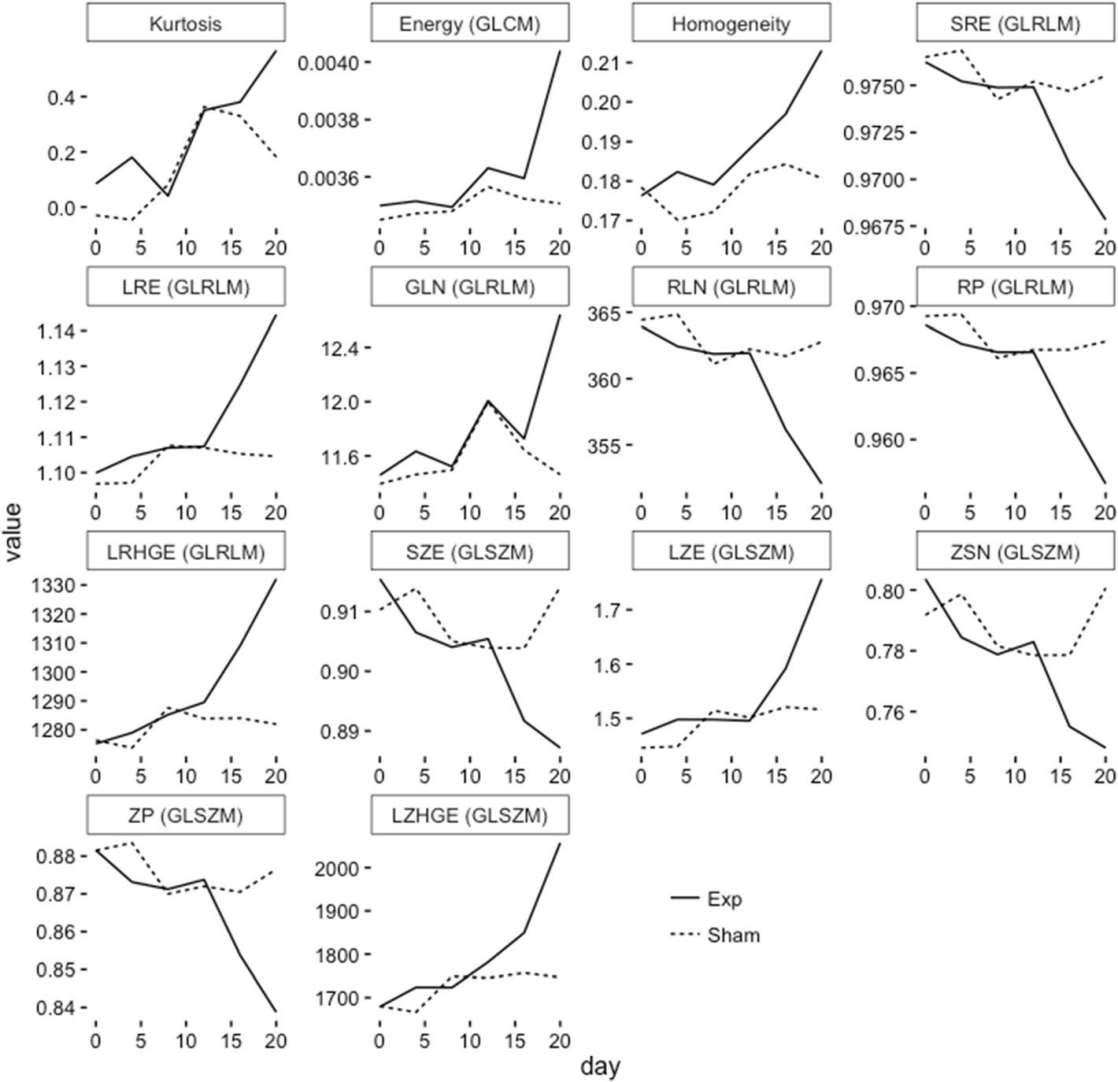
Set of features which change significantly after the injection of tumor cells at day 0, but not in the control group after sham injection of PBS

A selected set of those features is shown in Fig. 4. Five features correlated significantly in the sham group:

- GLRLM: LGRE, SRLGE
- GLSZM: LGZE, GLV, ZSV

However, none of them were also significantly correlated in the experiment group.

**Fig. 5:**
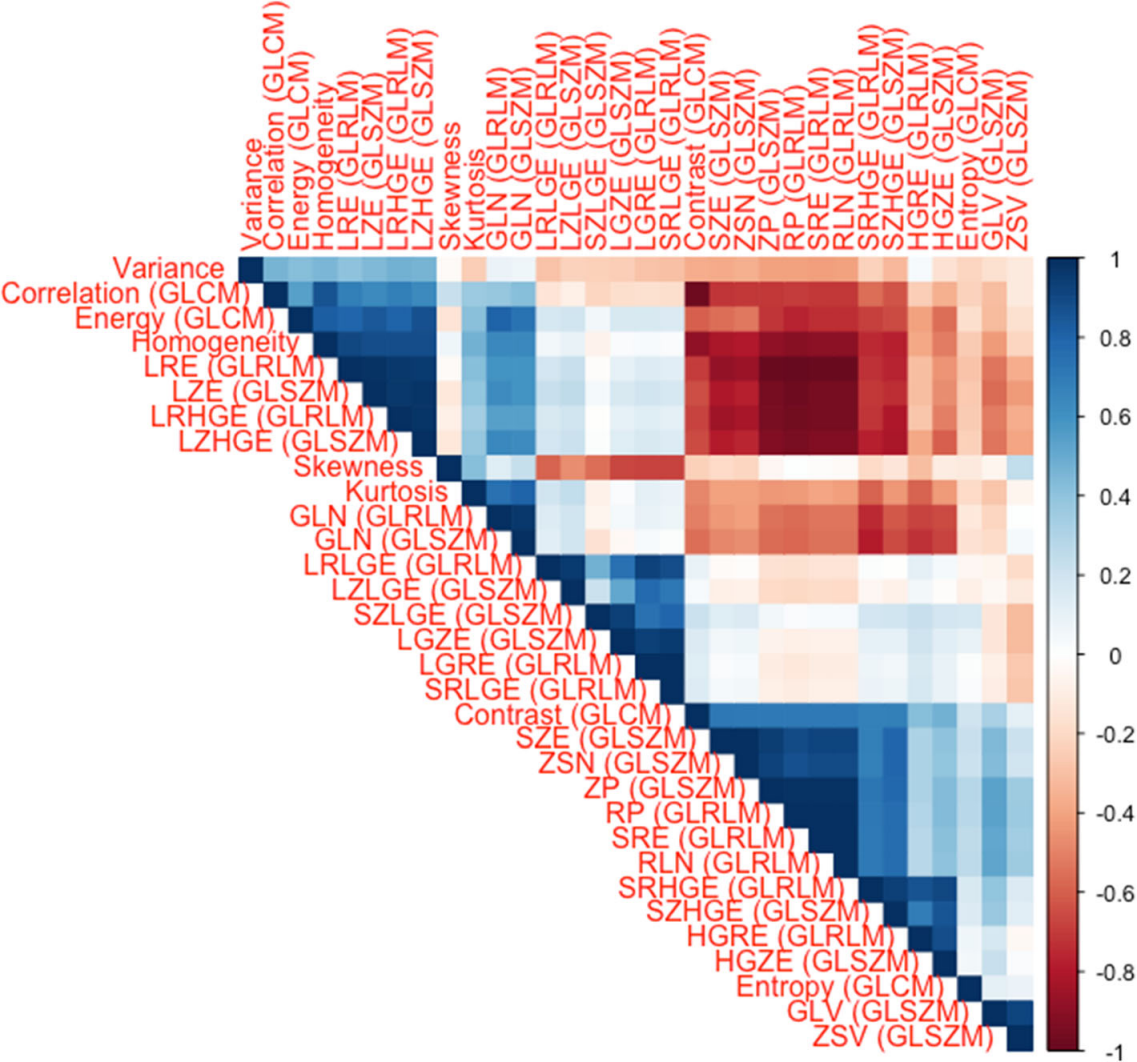
Correlation matrix showing the redundancy of many features. However, some clusters of independent features can be identified. The Pearson correlation coefficient R is color-coded according to the scale on the *right*

Hierarchical clustering revealed three distinct, independent clusters of features as depicted in Fig. 5. The most representative features, i.e. the ones with the smallest variance were Energy, SRE (GLRLM), and GLN (GLSZM).

## Discussion

In the present study, we examined whether changes of texture features may herald metastases in liver MRI before they can be appreciated visually. We found three independent features, one derived from each of the gray-level matrices, which exhibit a linear correlation before the metastasis is visible to the naked eye, and several co-dependent features. Thereby, we showed that texture analysis is able to detect subtle changes of parenchymal changes before a morphological lesion is visible, which may significantly enhance tumor detection rates in liver imaging.

Recently published studies have demonstrated that texture analysis can distinguish or classify benign and malignant lesions in various organs and tumors, for example in glioma/glioblastoma [25, 26], breast [27], lung [28], stomach [29], prostate [30], or liver lesions [15, 31]. Another recent focus of texture analysis has been the assessment of therapy response, e.g. in advanced ovarian and primary peritoneal cancer [32], or the prediction of lymph node metastasis from a radiomics analysis of the primary tumor [33]. However, to the best of our knowledge, no study has so far investigated the feasibility to detect cancerous lesions directly in the target tissue before they appear visible to the human reader. In our opinion, this application logically follows from the common denominator of the abovementioned studies: quantifying underlying histological changes in tissue below the resolution of the given modality or protocol.

Leonard et al. [34] as well as Adam et al. [35] showed that an increased number of patients with liver metastases undergo potentially curative hepatic resection because of recent progress in neoadjuvant chemotherapy. Still, optimal surgical planning depends on exact knowledge of the number and location of all liver lesions. Recently published studies showed high diagnostic accuracy for the detection of liver metastases in modern imaging modalities such as MRI and PET/MRI [36]. However, about two-thirds of the patients who have undergone liver resection for colorectal metastases suffer from recurrence within 18 months [37]. One reason is probably the fact that small liver metastases below or close to the resolution limit of the current imaging modalities on pre-surgery imaging are missed and, therefore, not taken into account. ^18^F-fluorodeoxyglucose (FDG) PET/CT is of little adjunctive value in these cases due to the high background glycolytic activity of the liver [38].

Texture analysis may thus be a new objective method to detect these lesions and improve post-surgery outcomes and disease-free survival interval. On the basis of the current data, it is not possible to determine whether the textural changes are a result of the metastatic cells themselves or rather a reflection of reactive changes in the surrounding liver parenchyma. Interestingly, features derived from all three gray-level matrices appear to be influenced by the metastatic growth, which could be an indication for the destruction of liver acini (alteration of co-occurrence and size-zones) or the tumor neovascularization (run-lengths of vessels). Further research in this area may be desirable as understanding the exact mechanism may aid for example in development of better MRI sequences suitable for texture analysis.

Our study has several limitations that need to be acknowledged. First, although the images were prospectively acquired, ROI definition had to be performed retrospectively after a suitable lesion was identified at the study end. Furthermore, we have only evaluated single slices. Because we aimed for a maximum in-plane resolution, the sequence was not acquired with isotropic voxel size and respective slice gaps. Performing three-dimensional texture analysis would have either required interpolation (which has been shown to confound the analysis [39]) or a lower resolution. Thus, we believe that three-dimensional analysis would not have added value to our results or altered our conclusion. Second, we only computed a limited set of features. We chose to do so, instead of analyzing a larger set of multiple hundred or thousand (compound) features, because the selected features have repeatedly been found useful in the analysis of medical images [15, 28, 30, 40] and robust against variations between scanners and protocol parameters [41], especially after normalization [20]. Moreover, our small sample size did not allow us to use multiparametric/hybrid imaging or machine learning algorithms to assess the usefulness of such a large number of features, which is the third main limitation. However, adhering to the 3R-principle (“Replace-Reduce-Refine”) [42], the small number of animals was a deliberate effort to keep the suffering of animals as low as possible. Hence, we think that the next step after this small pilot study should not be more experiments in animals, but rather a longitudinal study directly in human patients, e.g. a cohort at risk for hepatic (colorectal cancer) metastases. Until further validation in human studies, the implications of this work for patient care remain unclear.

In conclusion, we found in our small pilot study that texture analysis of MRI data may have the potential to detect liver metastases at a sub-resolution level, before they become visible to the human eye.

## Abbreviations

GLCM: Gray-level co-occurrence matrix
GLRLM: Gray-level run-length matrix
GLSZM: Gray-level size-zone matrix
MRI: Magnetic resonance imaging
PBS: Phosphate-buffered saline
ROI: Region of interest
